# Home- and Community-Based Interventions for Physical Activity and Early Child Development: A Systematic Review of Effective Strategies

**DOI:** 10.3390/ijerph191911968

**Published:** 2022-09-22

**Authors:** Samantha Moss, Xiangli Gu

**Affiliations:** 1 Department of Kinesiology, State University of New York at Cortland, Cortland, NY 13090, USA; 2 Department off Kinesiology, University of Texas at Arlington, Arlington, TX 76019, USA

**Keywords:** early childhood, physical activity, obesity, review, home environment, development

## Abstract

This systematic review examined the effects of home/family and community-based interventions on physical activity (PA) and developmental outcomes in early childhood. A search strategy was employed using four electronic databases (Academic Search Complete, CINAHL Complete, MEDLINE, and SPORTDiscus). Interventions investigating weight status (i.e., BMI), physical activity, sedentary behavior, and/or motor proficiency that took place in home, family, or community settings were assessed. Studies were eligible if they were peer-reviewed, available in English, published between 2011 and 2021, and if samples consisted of healthy young children (2–5 years old). There were 24 studies retained (8351 participants) spanning from the United States (n = 12), Australia (n = 3), Canada (n = 2), Switzerland (n = 2), Finland (n = 2), Netherlands (n = 1), and other Eastern European countries (n = 2). There were 19 studies that incorporated home/family-based approaches and 14 studies that incorporated community-based approaches. Studies ranged in intervention duration from 6 weeks to 24 months. It suggests that improving PA participation in young children was especially challenging to solicit improvement (only 25% of all studies found significant improvement in PA after intervention). Distributing educational material to parents/families, consistent, direct contact with parents, and encouraging community engagement were identified as effective strategies to promote physical activity, healthy weight status, and motor skills in young children.

## 1. Introduction

The unfavorable trend of childhood obesity (i.e., excess body mass) has more than tripled over the last forty years, increasing the prevalence of overweight children to nearly 20% [1,2]. One way to measure obesity is through calculating body mass index (BMI) by dividing body weight in kilograms by the square of body height in meters (k/m^2^) [3]. Using growth trajectories, researchers predict that 57.3% of children today will become obese by 35 years of age [4]. Not only will comorbidities of obesity include metabolic consequences (i.e., Type 2 Diabetes, cardiovascular disease, hypertension), but psychological effects may continue to persist into adolescence and adulthood [5]. Obesity prevention at an early age has been recommended [6]; however, early screening and intervention to deter the long-term consequences of childhood obesity are not sufficient among toddlers and preschoolers (2–5 years old).

It has been shown time spent in physical activity (PA) and sedentary behavior (i.e., screen or sitting time) are important determinants of weight status and healthy development in early childhood [7]. A previous systematic review reported that only 54% of young children (ages 2–6) met the National Association of Sport and Physical Education’s PA guideline of 60 min of both structured and unstructured PA per day [8]. Accordingly, only 35.6% of children aged 2–5 met the recommended guideline of only 1 h per day of screen time [9]. Child development is the process of evolution and growth from infancy into independent adulthood and can be categorized into four categories: motor skills; speech and language; social and personal activities of daily living; and performance and cognition [10]. Fundamental motor skills (FMS) are an important parameter of child development and can be assessed to indicate the presence of developmental delay. FMS are considered the building blocks of proficient movement and consist of basic movements that are important in physical development [11]. Achieving competency in FMS during early childhood also serves as a determinant of childhood obesity and has been reported to be significantly related to PA levels in young children [12,13]. Various intervention strategies aimed to increase PA, decrease sedentary behavior, and improve FMS to deter obesity are well-documented [14,15,16,17,18,19], however, most of the available literature only targets childcare and/or preschool settings. Caution should be taken when interpreting findings due to conflicting results, varying durations and settings of the intervention, and methods of assessments.

Sallis et al. [20] introduced a social-ecological model that illustrates the multi-facet nature of physical activity and human behaviors. In such, each level consists of a different environmental component with the most proximal level representing individual characteristics, followed by social environment, physical environment, and lastly geographical environment. Resources and opportunities to engage in PA and FMS practice are largely dependent on social-demographic (i.e., race/ethnicity, socio-economic status, gender) and environmental factors [21]. For instance, many studies have investigated associations of center-based environments on childhood health outcomes [22,23,24]. There is, however, less conclusive research on intervention strategies targeting children’s home/family or community, even though emerging research shows parenting behaviors greatly affect children’s healthy development through role modelling and conducive home environments [25]. Home/family-based interventions have been seen as a potentially viable route for obesity prevention programs to induce long-term, sustainable outcomes [26]. Center-based interventions may not solicit sustained changes in PA and child health, so it is necessary to determine what environmental factors are salient for continual PA engagement and positive development in early childhood from the available literature for future policy creators and practitioners. Supported by the social-ecological perspective, various components of the home/family environment (i.e., parental support and modeling, affordances/opportunities for PA, inside space and availability of toys, and built environments) may be correlated with PA participation and early childhood developmental outcomes, such as FMS [27,28,29].

Furthermore, previous systematic reviews and meta-analyses [30,31] suggest that home/family-based intervention strategies may be more effective for obesity prevention and child health, however, very limited strategies or recommendations were especially focused on PA and FMS promotion (i.e., lack of actionable strategies for practitioners). Knowlden and Sharma [31] also mentioned that parents’ educational sessions were the primary modality for intervention delivery, and only nine studies were focused on home/family settings (less than one-quarter of those included studies conducted home visits). Over the past 10 years since the publication of this review, advances in technology, methods of assessment, and longitudinal research has emerged, requiring a more current review on the efficacy of home-based interventions in early childhood. Thus, the purpose of this systematic review was to examine and analyze the effects of home/family- and community-based interventions on weight status (i.e., body mass index [BMI]), PA (i.e., moderate-to-vigorous PA [MVPA], sedentary behavior) and developmental outcomes (i.e., FMS) in early childhood (2–5 years old). This systematic review provided actionable strategies and recommendations for implementing effective and comprehensive interventions in early childhood (i.e., home and community settings) and further expose the salient social-ecological environmental factors that should be targeted for long-term obesity prevention and child development, respectively.

## 2. Methods

This systematic review was not registered but followed the Preferred Reporting Items for Systematic Reviews and Meta-Analysis (PRISMA) guidelines to report the searching process and strategies [32]. The flow diagram of excluded articles in the review process was provided in the results section and a PRISMA checklist has been provided in the Supplement File Table S1. The review protocol may be available upon request.

### 2.1. Search Strategy

Using a search strategy developed from a previously published approach shown to be sensitive for identifying systematic review [33], four electronic databases were comprehensively searched (Academic Search Complete, CINAHL Complete, MEDLINE, and SPORTDiscus) with full text. The search strategy focused on four components including participants, study design, intervention, and outcomes. Specifically, the following key terms were utilized in the searching process: “home-based interventions” OR “family-based interventions” OR “community-based interventions” AND “physical activity” OR “sedentary behavior” OR “motor skills/development” OR “obesity” OR “BMI” AND “early childhood” OR “toddlers” OR “children” AND “home environment” OR “social environment” OR “physical environment” OR “parenting behaviors”. Limiters were used for age (including only early childhood), peer-reviewed, and English availability. Two reviewers independently reviewed the results from the initial search of the abstract and then the full paper. The consensus and agreement were reached through discussion when opinions differed between two reviewers.

### 2.2. Eligibility Criteria

Studies were only included if there was a home/family- or community-based intervention with the intended related outcomes. Studies were conducted in a school setting, were not included in the review. Studies that employed a randomized controlled trial with and without control groups were considered as one of the selection criteria. Peer-reviewed articles published from 2011 onward, with full text availability in English were included. Intervention studies focused on typically developing children between the ages of 2–5 or parent–child dyads were included. Articles including interventions that were center or school-based, exclusively, were not retained. Citations were downloaded into Microsoft Excel and EndNote where duplicate articles were removed. Articles were first reviewed by their title and if retained, by abstract. Disagreements were resolved through discussion with included authors.

### 2.3. Data Extraction

Data extraction was completed by one reviewer and checked for accuracy by another graduate student and one faculty mentor. Information was displayed in a standardized Excel table regarding study characteristics (i.e., year, location, number of participants, average age, study design), intervention protocols, and results. Studies were categorized based on intervention setting (i.e., home- and family-based or community-based). For clarity, home/family-based settings were defined as approaches targeting the physical home or parenting routines, while community-based settings refer to approaches that utilized group settings (with other parents or children) or used community resources as an intervention tool. Outcome variables were also extracted and accurately coded into either PA, sedentary behavior/screen time, BMI/weight status, or motor skill proficiency.

### 2.4. Quality Assessment of Studies

To assess study quality, the Effective Public Health Practice Project Quality Assessment (EPHPP) Tool was employed [34]. The EPHPP (accessed on 12 April 2022; http://www.city.hamilton.on.ca/phcs/EPHPP/) is a generic tool used to assess a variety of intervention designs including randomized control trails (RCTs), repeated measures (pre-and post-) and case–control studies. This tool has been recommended for evaluating effectiveness of systematic reviews and construct validity [35]. This tool assesses six domains including, selection bias, design, confounders, blinding, data collection methods, and withdrawals and dropouts. Studies are rated as *strong*, *moderate*, or *weak* for each item based on specific criteria. If there are no reports of these specified items in the study, a grade of weak is given. Afterwards, studies with at least four strong ratings throughout and no weak ratings are considered strong; studies with less than four strong ratings throughout and one weak rating are considered moderate; studies with two or more weak ratings are considered weak.

### 2.5. Effect Measures

Effect size (ES) was calculated for each study and represented as the standardized mean difference. Some studies did not provide sufficient results for this to be calculated. Results are interpreted as small (ES ≥ 0.2), moderate, (ES > 0.5), large (ES > 0.8), or very large (ES ≥ 1.3) [36].

## 3. Results

### 3.1. Study Selection and Characteristics

**Figure 1:**
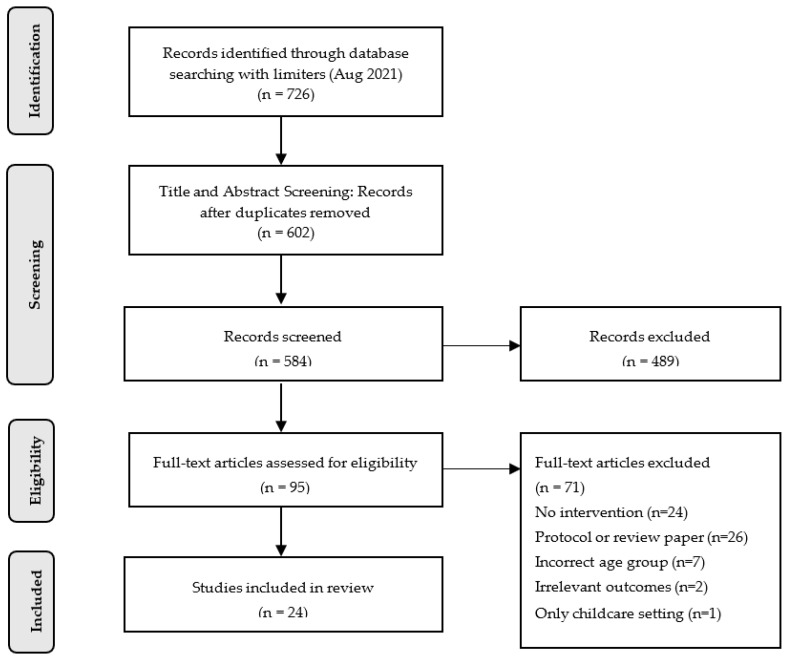
PRISMA Flow diagram of excluded articles throughout the review process.

The initial search yielded 726 articles. Through title and abstract screening, 602 remained. After thoroughly screening the full-text, 24 articles were included in the data analysis. The details of our analysis procedures are shown in Figure 1.

There was a total of 24 studies with 8351 participants included in this review. Nearly 80% of included studies implemented a home/family-based approach (n = 19) and 42% of those studies (n = 8) also incorporated aspects of community-based approaches in their intervention. Among included studies, majority were conducted in the United States (n = 12), Australia (n = 3), Canada (n = 2), Switzerland (n = 2), Finland (n = 2), Netherlands (n = 1), and other Eastern European countries (n = 2). The duration of these interventions ranged from 6 weeks to 24 months. The 24 studies were categorized by intervention approach (home/family-based or community-based) and described in detail below.

Regarding the quality and risk of bias assessment, only two articles showed a rating of “strong” in the global rating category. Most commonly, articles were not effective in blinding participants and suffered from selection bias in their recruitment phases, reflecting grades of “moderate” or “weak”. These results are presented in Table 1.

### 3.2. Effect Estimates of Studies

Studies that provided sufficient information were included in the effect size calculation (Table 2). Less than 1/3 of studies yielded an effect size of at least moderate effect (>0.5) in their measured outcomes. Three studies did not yield enough information for calculation.

### 3.3. Effects of Home/Family-Based Interventions on Outcomes

As shown in Table 3, only three out of the 19 studies that investigated the home/family-based intervention on PA outcomes found significant improvements after the intervention [37,38,39]. These studies were 2–6 months long in duration and disseminated educational materials and interactive games to families [37,39] and scheduled individual discussions with parents [38]. Out of the four studies that investigated motor skill outcomes in a home/family setting, three found significant improvements after intervention from providing educational material and actively staying in contact with parents [40,41,42]. Most of the studies found the home/family-based strategies, such as home visits and providing educational material to the parents, had significant impacts on sedentary behaviors and weight status [38,40,43,44,45,46,47,48,49,50,51,52].

Specifically, seven studies consistently noted that facilitated physical home visits may have significant impacts on BMI and screen-based sedentary behaviors with moderate-to-strong effect sizes [43,44,46,49,50,51,52]. However, there are inconclusive findings regarding the impact of home visits on PA engagement during early childhood. All studies using a home visit approach found no significant changes in PA (light PA, MVPA) and most of them used accelerometry as the assessment tool among young children. Tomayko, Prince, Cronin and Adams [51], for example, implemented a two-year long intervention targeting Native American preschoolers in Wisconsin (3–5-year-old children). The intervention was designed to be delivered in two separate formats during the first year: in-home mentoring (n = 75) or by mail (n = 75) but both groups received the same intervention materials. The second year of this intervention served as a maintenance year with continued support. During the home visits, mentors implemented culturally tailored lessons, activities, and resources designed to improve activity, screen time and weight status. Visits would occur once a month (12 total) for 60 min. The mail group would get the same content, only delivered to them non-face-to-face. There were no significant changes in BMI, physical activity behaviors, and screen time between groups (*p*s > 0.05), but significant improvements were reported within groups. Children improved in BMI and screentime (*p*s < 0.05), but not in PA.

The remaining studies did not have physical home visits within the intervention protocol, but did attempt to incorporate parental education, involvement, and/or engagement through disseminating materials, hosting discussions and informational group meetings, facilitating messages or emails to families/parents, or participating in activities with children in their interventions. However, two studies found no significant intervention effects on PA, sedentary behavior, BMI, or motor skills [53,54] by implementing similar methods of sending informational flyers/newsletters to parents. Studies that adopted an approach using direct contact to parents through messaging, calling, and individual meetings found improvements in PA, sedentary behavior, BMI, and motor skills after the intervention [38,41,48]. Specifically, Quattrin, Roemmich, Paluch, Yu, Epstein and Ecker [48] implemented a home/family-based intervention for promoting healthy weight status by providing parents a total of eight phone calls over the course of 12 weeks. The intervention group received greater emphasis on parenting behaviors and strategies to promote healthy modifications within their home during these calls compared to the control group. Keita et al. [55] also incorporated a similar approach of facilitating parental contact and found improvement in screentime (*p* < 0.01) but conversely found PA engagement to decline (*p* < 0.05) from pre- to post-assessment.

Other studies only disseminated materials to parents and did not incorporate individualized messaging or meetings with parents and only found significant improvements in sedentary behavior, BMI, and motor skills. Puder, Marques-Vidal, Schindler, Zahner, Niederer, Bürgi, Ebenegger, Nydegger and Kriemler [40] for example, found that just sending home materials related to physical activity or nutritional cards/brochures was not sufficient to change children’s PA compared to the control group; improvements in screentime was reported within the intervention group from pre- to post-assessment. Similarly, Knowlden and Sharma [45] employed a web-based approach containing interactive discussions, worksheets, and audiovisual presentations in a randomized controlled design for eight weeks. Screentime was seen to significantly improve (*p* < 0.01) but not physical activity (*p* > 0.05). A study by Zask, Adams, Brooks, Hughes, Zask, Adams, Brooks and Hughes [42] had parents receive monthly four-page newsletters containing tips for active playing to encourage playing in their home and found significant improvements in BMI z-scores and movement skills (*p*s < 0.05). Latomme, Cardon, De Bourdeaudhuij, Iotova, Koletzko, Socha, Moreno, Androutsos, Manios and De Craemer [47] found improvements in screentime after the 24-week intervention from sending newsletters, tip cards, and informational posters to parents.

### 3.4. Effects of Community-Based Intervention on Outcomes

There were 14 studies that implemented community-based approaches as part of their intervention protocol (Table 4). Only three studies found significant improvements in PA measures after the intervention by utilizing interactive activities by community members (i.e., social maps and scavenger hunts) and educational group sessions [38,56,57]. One study conducted by Walton et al. [58] had parents attend a two-hour group lesson/week for nine weeks and found no significant improvements in PA, sedentary behavior, or BMI.

Three studies promoted community participation and resources but found conflicting results towards PA engagement [41,56,59]. For instance, Davison, Edmunds, Wyker, Young, Sarfoh and Sekhobo [59] distributed a community calendar to families along with additional knowledge regarding physical activity and screen time and found children were more likely to watch less than two hours of TV per day, play outside, and meet the national physical activity recommendations (*ps* < 0.05). van de Kolk, Gerards, Harms, Kremers and Gubbels [56] distributed a social map outlining opportunities to engage in PA for young children within their own communities and found significant improvements in PA as well (*p* < 0.05). Trost and Brookes [41] implemented an FMS-based intervention via mobile application. In the app, users are encouraged to share their accomplishments with other community members but found no significant improvements in PA.

The remaining studies implemented group or peer engagement, and some found significant improvements in sedentary behaviors [38,40,51,56] but the findings are mixed. Studies that were successful in improving sedentary behavior required parents to attend minimal group sessions (i.e., one 30 min session per month) whereas the studies that were unsuccessful required parents to attend a series of lectures or group meetings multiple times per week or month [54,58].

There were approximately 42% of studies did not find significant improvements in BMI despite most studies did use objective measures to calculate BMI [38,53,54,56,58]. One study, in particular, did find significant BMI improvement and was the only intervention that utilized peer engagement within children, not just parents [49]. While parents were attending a 90-min education session, children would participate in group-based activities (*p* < 0.01). Zask, Adams, Brooks, Hughes, Zask, Adams, Brooks and Hughes [42] embedded an FMS-based curriculum within their sample and found children in the intervention group had significantly better BMI post-test (*p* < 0.05). On the other hand, other studies focused on socializing and educating parents in groups and did not facilitate any child activities, but found significant BMI improvement in young children [40,48,51,60,61].

Studies improved young children’s motor skills when researchers facilitated in-person workshops or lessons that educated parents on PA and FMS [40,42,61]. However, employing similar strategies for enhancing parental education, Bonvin, Barral, Kakebeeke, Kriemler, Longchamp, Schindler, Marques-Vidal and Puder [53] did not yield significant improvements in motor skills (*p* > 0.05).

### 3.5. Summary of Identified Recommended Strategies

The tables below display the synthesized recommended intervention strategies for future researchers and practitioners for promoting physical activity, reducing sedentary behavior/screen time, promoting healthy weight/BMI and promoting motor skills, respectively. Outlined in Table 5, six studies have been identified as providing effective strategies for PA promotion in early childhood. Distributing education material to parents/guardians, maintaining direct contact with parents throughout the duration of the study, promoting community resources (i.e., opportunities for activity within the community), encouraging community engagement through scheduling group sessions with parents/guardians, and implementing a peer education component were all shown to elicit positive PA outcomes in early childhood.

Effective strategies for reducing sedentary behaviors and screentime are outlined in Table 6. Eleven studies were included in this table with stratigies comprising of scheduling physical home visits to each participant for either pre-assessment measures or as a protocol in the intervention, distributing educational materials to parents/guardians, maintaining direct contact with parents and families, enducing parent participation in activities with their children, promoting community resources available to residents, and encouraging community engagement through scheduling group sessions.

Table 7 demonstrates effective strategies for promoting healthy weight status/BMI measures which included the citation of 12 studies. Scheduling home visits, distributing education materials to parents/families, maintaining direct contact with parents, encouraging community engagement and facilitating peer education sessions elicited positive changes in children’s BMI and/or weight status.

Lastly, effective strategies for the enhancement of motor skill development is displayed in Table 8. Four studies were included in this table and the strategies include distributing educational material to parents and families, maintaining direct contact with parents throughout the duration of the intervention, promoting community engagement, and facilitating peer education.

## 4. Discussion

This review aimed to examine and analyze the effects of various home/family- and community-based interventions on PA behaviors and developmental outcomes in early childhood. This review highlights home/family, and community-based intervention strategies that were effective in reducing unhealthy weight, promoting PA, reducing sedentary behavior (i.e., screen time), and enhancing FMS. Studies targeting home/family and community avenues are gaining interest and have been shown to be a viable avenue to pursue in terms of early childhood obesity prevention and activity promotion. Research supports the positive relationships among PA, sedentary behavior, FMS with weight status in early childhood [12,62] thus intervening at an early age, in a comprehensive environment, is critical for long-term health.

Overall, most studies received a global rating from the risk assessment as weak due to selection bias, not controlling for confounding variables, and lack of blinding. Of course, with an exercise or obesity prevention intervention, blinding participants to the treatment is almost impossible. It also seems challenging to obtain and sustain participation throughout studies of this nature, thus, it is recommended to implement a fidelity framework to uncover strategies for increased adherence and sustainability for the PA and health promotion intervention. The RE-AIM framework may be a useful theoretical guide for future implementation to reach individual and organizational level effects [63]. Incorporating this framework could solicit useful information on the reach, efficacy, adoption, implementation, and maintenance of a randomized controlled study or other evidence-based practices.

Studies that were allocated into the home- and family-based intervention category focused on strategies including physically visiting the home of the families, enhancing parental knowledge, involving parents in activities, and/or engaging parents through means of communication. Studies that showed more positive impact from their intervention all embedded educational tactics throughout the duration of the study to parents in home settings. These strategies consisted of informational newsletters and brochures, interactive and informational toolkits/activities, and individual education-led discussions. Educating parents and families on child health-related outcomes, such as physical activity recommendations and guidelines, different affordable and fun ways to be active together, consequences of excess sedentary behavior, motor skill enhancement activities, and weight-related content should be prioritized to be disseminated to parents and families. Studies did use opposing approaches in getting these materials across, such as providing informational (news) letters or using fairy tales and tip cards to display the information. Taking a modern approach may be a more feasible way to achieve this for large study samples. For instance, some studies have found success in using technology (i.e., Facebook groups, mobile messages) as a tool for communication and material/knowledge disbursement to parents because of the convenience of accessing the information from virtually anywhere [64,65]. Most studies included in this review used traditional methods of disbursing knowledge-based content (i.e., printed materials delivered through mail or children would take materials home from school). Two studies, however, employed technological methodology; a web-based approach [45] and use of a mobile application [41]. In both studies, adherence to the program was positive through technology-facilitated parent–child interaction, but no significant effect on physical activity support. Perhaps, if there was a parental knowledge aspect incorporated into this intervention, parental support for physical activity could have yielded significant improvements.

Community engagement was also found to be a viable and effective strategy that yielded significant effects on all outcomes. Interventions that target enhancing social support and group settings, for both children and parents, may improve children’s behavioral health outcomes [66,67]. The findings of this review provide valuable insights that interventions aimed to promote healthy weights and behaviors (e.g., PA, sedentary behavior) may consider parents as agents of change. It’s important to also acknowledge the differences in effective methodology from the intended outcomes. For instance, home visits were only effective strategy on changing sedentary behaviors/screen time and BMI, but not on physical activity or FMS among children. It suggests that the dose of home visits (e.g., frequency, duration) in the intervention may be an important factor to be considered in the future as its effect was significant on other health outcomes in the most of reviewed studies. If a family does not regularly participate in physical activity, it would be challenging to incorporate a new habit rather than modify what is already being done (e.g., reducing the amount of time spent watching TV). It is important to further understand how habits affect behaviors for sustained engagement, and how habits/behaviors are formed, broken, and changed during health intervention research [68].

Although studies varied in design and delivery, the purposes of each remained consistent throughout, which was to test the effectiveness of their respected intervention towards child health outcomes. However, the greatest difference among all studies was the duration and frequency of intervention delivery (6 weeks-24 months). Two studies employed home visits in their 24-month intervention and found significant impacts on children’s sedentary behaviors and weight status, but not physical activity participation [51,52]. The frequency of the home visits was comparable (one 60-min visit per month for 12 months vs. eight 60-min visits within 24 months, respectively). In the 6-week study, home visits (eight 60-min visits) results showed sedentary behavior to be significantly improved as well [46]. Those findings support the home visits serve as the most salient intervention component regardless of the duration of the intervention. More importantly, the delivery and degree of changing the child’s home environment (i.e., assessing and modifying familial routines at home, providing interactive toolkits) may elicit greater influences on children’s sedentary behaviors and weight status. Thus, it may be helpful for future studies to provide clarity on intervention dosage (i.e., frequency and duration) effects on children’s weight-related outcomes to underline efficient and feasible intervention lengths. It suggests that improving PA participation in young children was especially challenging to solicit improvement regardless of the intervention duration (only 16% of studies found significant improvement in PA after intervention). In these successful programs, group interaction among parents through lectures or discussions were consistently implemented strategies which are also recommended for future PA and health-promoting interventions among your children.

There were some studies that did not yield any significant changes in child health outcomes, despite incorporating both home/family and community-based approaches. One study explains that the possible reason for that may be the intervention was government-led, rather than investigator-led [53]. Other studies attribute their lack of significant impact to reasons including not adequately equipping parents and families with enough education on weight-related topics, and parents having inaccurate perceptions of their children’s overweight status prior to the intervention starting [54,58]. It may be important for future studies to have researchers thoroughly involved in the implementation and delivery of the intervention, rather than rely on a government coordinator, to ensure proper knowledge and training are being provided to participants and research personnel. It also may be beneficial for researchers to gauge parents’ knowledge of their child’s obesity-related behaviors prior to the start of the study for a baseline, to help provide parents with an accurate perception of their child to better enhance their knowledge throughout the study.

Several studies did investigate long-term effects through follow-up testing (6 months–24 months) [38,43,44,45,51,52,54,56,58]. Including follow-up measures in an intervention study can expose the sustainability of different strategies that were incorporated to determine lasting effects (i.e., increasing PA, reducing sedentary behaviors). Haines, Douglas, Mirotta, O’Kane, Breau, Walton, Krystia, Chamoun, Annis, Darlington, Buchholz, Duncan, Vallis, Spriet, Mutch, Brauer, Allen-Vercoe, Taveras, Ma and on behalf of the Guelph Family Health [44] and Wen, Baur, Simpson, Rissel, Wardle and Flood [52] found significant sustained improvements in BMI at their 6-month and 24 month follow-ups, respectively. However, Walton, Filion, Gross, Morrongiello, Darlington, Randall Simpson, Hou and Haines [58] and van de Kolk, Gerards, Harms, Kremers and Gubbels [56] found no significant long-term effects on BMI in children based on the follow-up assessment. It was observed that both Haines et al. and Wen et al.’s studies support the sustained long-term effects on body weights as both studies incorporated home visits into their interventions. It suggests that changes occurring in the home environment, particularly focusing on parents’ perception or behavioral education may prompt a greater influence in change of behavioral habits which may have long-term impact on weight management skills/knowledge. In terms of PA, only one study Knowlden and Sharma [45] support the sustained improvements in PA and the intervention was focused on delivering the educational materials through an interactive web-based approach. This study provides preliminary evidence that technology may be a more effective intervention approach compared to other traditional PA interventions during childhood.

Given the heterogeneity in these reviewed assessments to measure physical activity, sedentary behaviors, weight status, and motor skills, generalizing findings is challenging. Some studies used objective measures (i.e., accelerometers to measure physical activity intensity levels and sedentary behavior, measuring children’s height and weight directly) while others relied on parent-report measures (i.e., surveys). The majority of studies that measured physical activity did employ the use of objective approaches, such as accelerometers or pedometers (12/20), which makes the findings of their studies more robust. From the developmental perspective, young children’s daily movement and activities are under supervision of parent’s guidance and the types of PA children engage in may easily be changed throughout the home- and community-based intervention instead of their intensity levels of PA captured by accelerometry. This may be a possible reason why only very few studies supporting the home/family-based intervention have a significant impact on PA in this age group. It’s important for future studies, especially using a home/family-based approach, to incorporate both objective (assessing PA intensity levels) and parent-report measures (assessing types of activities/movements) to comprehensively assess effectiveness. It’s also recommended for future studies to plan for follow-up measures to assess long-term effects and fidelity.

Another important finding of this review is the lack of studies targeting specifically toddler-aged behaviors and development outcomes (2–3 year old children). A recent systematic review and meta-analysis assessed 2–6 year old children’s accelerometer-measured sedentary behaviors but results were grouped together for toddlers and preschoolers, making it difficult to dissect the different age groups’ specific findings [69]. In the current review, some studies included both toddler and preschool aged children, but also did not separate findings by age group, making tailored and age-group-specific conclusions unclear. In early childhood, the evidence suggests that adequate FMS proficiency and PA are positively correlated with cognitive outcomes [70,71,72,73], so interventions conducted within the toddler age group could be more beneficial and effective than starting interventions later in childhood. Future studies are encouraged to exclusively target toddler-aged groups when assessing physical activity and motor skills for a clearer picture of this vulnerable and understudied group.

Throughout searching for this review, opposing verbiage was a theme that was noticed. For example, some studies would proclaim the study was “home-based” but the approaches differed in being delivered in the home environment physically, or measuring aspects of the home environment (i.e., family integration, parenting practices). Studies also used terms such as “family-based”, “family-integrated” “community-based” and even “school-based” when the aims of the interventions were still targeting change of familial routines/habits and including parents in the intervention activities and protocols. This review highlights the need for more consistent verbiage throughout home- family- and community-based interventions for a more comprehensive understanding of the setting and delivery of the treatment.

It’s important to include educational materials, specific to the study outcomes, to enhance parents’ familiarity and promote the benefits of physical activity, healthy weight status, and motor skill proficiency in young children. It’s also important to note that direct contact with parents and families was an effective method across all four outcomes as well, such as email, texting, phone calls, and face-to-face individual conversations with parents/families. These can all be achieved in a practical method. With the lingering effects of COVID-19, physical interaction between researchers and participants (or children) can still be challenging. This review supports that other modes of communication (paper copies of materials, virtual interaction through mobile devices) can also yield positive benefits as physical home visits do. Knowing the intrinsic role parents and home social environment play in childhood health and development, using methods of direct contact with parents to sustain engagement, assess progress, and encourage adherence proves to be essential intervention strategies. Parents play a major role in supporting the developmental trajectories of their preschool children, thus, including parents as directly as possible within the intervention may be necessary for future studies [74].

This review employed a search strategy including both toddlers and preschoolers to encompass a broad picture of early childhood health behaviors and development, which provides a great addition to the literature. This review also did not limit findings to just physical activity, but also correlates physical activity (i.e., BMI, sedentary behavior, motor skill proficiency) for a more complete assessment of effective intervention strategies and settings in early childhood. However, only studies published in English were included, limiting the possibility of other studies that may fit the inclusion criteria to be included. This review also was limited to searching four databases, as such, it is recommended for other or additional databases to be searched in future reviews.

For future interventions, it is recommended to incorporate a multi-level and multi-component intervention in home/community settings aimed to improve early childhood PA and health outcomes. Emphasizing parent education and engagement through various approaches (e.g., home visits, mobile messages, Facebook groups) is strongly recommended for sustained positive impact. Interventions embedded in parenting PA practices (i.e., improved household activity routines, reduced screen time) along with other educational components in home setting could facilitate a positive impact on healthy weight and behavioral management during early childhood.

## 5. Conclusions

Overall, this review highlights intervention approaches that were deemed effective in improving children’s physical activity, sedentary behaviors, weight status, and motor skill proficiency. Our findings indicate that using a comprehensive strategy, including producing and disseminating appropriate parental knowledge-based material, directly and frequently contacting parents/families for check-ins, encouragement, and transparent communication, and using group or community-based groups for parents and children are the most vital aspects to solicit improvements PA and developmental outcomes during early childhood.

## Figures and Tables

**Table 1:** Risk of bias assessment–EPHPP assessment tool.

	Domain Rating						
Articles	Selection Bias	Study Design	Confounders	Blinding	Data Collection Methods	Withdrawals and Dropouts	Global Rating
Barkin et al., 2013	Strong	Strong	Weak	Weak	Moderate	Moderate	Weak
Bonvin et al., 2013	Weak	Strong	Strong	Weak	Strong	Strong	Moderate
Davison et al., 2011	Weak	Weak	Moderate	Moderate	Strong	Weak	Weak
De Craemer et al., 2014	Moderate	Strong	Strong	Weak	Strong	Moderate	Moderate
Ftizgibbon et al., 2013	Strong	Strong	Strong	Weak	Strong	Strong	Moderate
Haines et al., 2013	Weak	Strong	Weak	Weak	Moderate	Strong	Weak
Haines et al., 2018	Strong	Strong	Strong	Weak	Strong	Strong	Moderate
Keita et al., 2014	Weak	Moderate	Weak	Weak	Strong	Moderate	Weak
Knowlden & Sharma et al., 2016	Moderate	Weak	Weak	Weak	Strong	Moderate	Weak
Koulouglioti et al., 2013	Weak	Moderate	Weak	Weak	Strong	Strong	Weak
Latomme et al., 2017	Moderate	Strong	Strong	Weak	Strong	Weak	Weak
Laukkanen et al., 2017	Weak	Strong	Strong	Weak	Strong	Weak	Weak
Puder et al., 2011	Strong	Strong	Strong	Moderate	Strong	Strong	Strong
Quattrin et al., 2012	Strong	Strong	Strong	Moderate	Moderate	Strong	Strong
Ray et al., 2020	Weak	Strong	Strong	Weak	Strong	Strong	Weak
Stark et al., 2014	Weak	Strong	Moderate	Weak	Strong	Moderate	Weak
Taverno Ross et al., 2018	Strong	Moderate	Weak	Weak	Strong	Strong	Weak
Tomayko et al., 2016	Weak	Strong	Weak	Moderate	Weak	Moderate	Weak
Trost & Brookes et al., 2021	Strong	Strong	Weak	Moderate	Strong	Strong	Moderate
Van de Kolk et a., 2019	Weak	Moderate	Strong	Weak	Strong	Moderate	Weak
Walton et al., 2015	Weak	Strong	Strong	Weak	Weak	Strong	Weak
Wen et al., 2012	Strong	Strong	Moderate	Moderate	Strong	Moderate	Moderate
Yin et al., 2012	Strong	Moderate	Strong	Weak	Moderate	Strong	Moderate
Zask et al., 2012	Moderate	Strong	Strong	Weak	Moderate	Strong	Moderate

*Notes. Weak* = there were no components explained to justify incorporating this domain; *Moderate* = some components were explained to justify incorporating this domain; *Strong* = this domain was accurately explained and accounted for. Global rating with 3 level scale: *weak* = 2 or more weak domains; *moderate* = 1 weak domain; and *strong* = no weak domains.

**Table 2:** Effect sizes of measured outcomes (n = 24).

Articles	Outcome Measures Effect Size			
	PA	SB/Screen Time	BMI	Motor Skills
Barkin et al., 2013			-	
Bonvin et al., 2013	0.19	0.08		0
Davison et al., 2011	-	-		
De Craemer et al., 2014	0.16			
Fitzgibbon et al., 2013	1.24	0.20		
Haines et al., 2018	0.65 to 0.64	−1.46 to 0.17	−1.30 to −3.54	
Haines et al., 2013		0.37	0.15	
Keita et al., 2014	0.43	0.40	0.05	
Knowlden & Sharma et al., 2013	0.04	0.02		
Latomme et al., 2017		0.11		
Laukkanen et al., 2017	0.10	0.21		
Puder et al., 2011	0.01	0.26	0.06	
Quattrin et al., 2012			0.42	
Ray et al., 2020	0.08	0.08		
Stark et al., 2014			−1.64 to −0.088	
Taverno Ross et al., 2018	0	0.03	0.04	
Tomayko et al., 2016	0.24	0.03	0.19	
Trost & Brookes, 2021	0.06			0.61 to 1.1
Van de Kolk et al., 2019	−0.19 to −0.24	0.21 to 0.33		
Walton et al., 2015	0.60	0.10	0.07	
Wen et al., 2012	-	-	0.22	
Yin et al., 2012			0.02 to 0.12	0.69 to 0.80
Zask et al., 2012			1.70	2.33 to 5.15

*Notes.* - = not enough information to calculate effect size. PA = physical activity; SB = sedentary behavior. Classification of effect size: small effect size (>0.2); moderate effect size (>0.5); and large effect size (>0.8); very large effect size (>1.3).

**Table 3:** Intervention effects of home/family-based intervention (n = 19).

					Outcomes			
**Author**	**Location**	**N (Age)**	Design	Intervention Protocol	PA	SB/Screen Time	BMI	Motor Skills
Bonvin et al. (2013)	Switzerland	648 children (M = 3.3 years)	9-month RCT	Informational flyers were sent to parents	X ^0^		X ^0^	X ^0^
De Craemer et al. (2014)	Belgium	472 children (M = 4.4 years)	24-week RCD	2 newsletters, 2 tip cards, and an informational poster was sent/presented to parents	X *			
Fitzgibbon et al. (2013)	United States	157 children (M = 4.5 years)	14-week RCT	1 newsletter/week were sent to parents A take-home music CD was sent home to parents	X ^0^	X ^0^	X ^0^	
Haines et al. (2018)	Canada	55 children (M = 3.0 years)	6-month RCT	2–4 home visits for 30–60 min each Behavior change emails were sent to parents	X ^0^	X ^0^	X *	
Haines et al. (2013)	United States	111 children (M = 4.1 years)	6-month RCT	For the first 16 weeks ○ 4 home visits ○ 1 phone call/month ○ 2 texts/week For the last 8 weeks ○ 1 text/week Educational materials were sent to parents		X *	X *	
Keita et al. (2014)	United States	39 children (M = 3.7 years)	4-month Prospective Design	1 set of written material/4 weeks were sent to parents 3 phone calls were given to parents A family exercise video was sent to parents TV monitor (to restrict children’s screen time) was delivered to parents	X * (-)	X *	X ^0^	
Knowlden & Sharma (2016)	United States	44 children (M = 5.2 years)	8-week Mixed between- subjects design	5 modules (10–15 min videos, newsletters, worksheets, emails) were delivered to parents through web-based methods	X ^0^	X *		
Koulouglioti et al. (2013)	United States	11 children (3–5 years old)	6-week Single group pre-post design	4 home visits for 1 h each		X *		
Latomme et al. (2017)	Belgium, Bulgaria, Germany, Greece, Poland, and Spain	2434 children (M = 4.7 years)	24-week RCD	2 newsletters, 2 tip cards, and an informational poster was sent/presented to parents		X *		
Laukkanen et al. (2017)	Finland	44 children (M = 6.1 years)	6-month RCT	2 phone calls were given to parents Individual discussions were held with parents	X *	X *		
Puder et al. (2011)	Switzerland	652 children (M = 5.2 years)	12-month RCT	Brochures, activity cards, and worksheets were sent to parents	X ^0^	X *	X ^0^	X *
Quattrin et al. (2012)	United States	96 children (M = 4.6 years)	12-week RCT	8 phone calls were given to parents One-on-one meetings were scheduled as needed			X *	
Ray et al. (2020)	Finland	802 children (M = 5.1 years)	4-month RCT	Informational letters, emails with videos/articles, bingo games, and fairy tales were sent to parents	X *	X ^0^		
Stark et al. (2014)	United States	18 children (M = 4.6 years)	6-month RCT	6 months 18 home sessions for 60–90 min each	X ^0^		X *	
Taverno Ross et al. (2018)	United States	49 children (M = 3.9 years)	10-week Single group pre-post design	90-min home sessions/week	X ^0^	X *	X *	
Tomayko et al. (2016)	United States	150 children (M = 4.0 years)	24-month RCT	1 home session/month lasting 60 min	X ^0^	X *	X *	
Trost & Brookes (2021)	Australia	34 children (M = 5.3 years)	8-week RCT	1 email and 1 text/2 weeks were sent to parents	X ^0^			X *
Wen et al. (2012)	Australia	667 children (Range: 2 years)	24-month RCT	8 home visits Individualized educational kits were sent to families	X ^0^	X *	X *	
Zask et al. (2012)	Australia	560 children (M = 4.5 years)	10-month RCT	1 newsletter and other written materials/month were sent to parents			X *	X *

*Notes.* RCT = randomized controlled trial, RCD = randomized cluster design, X * = significant positive effect at *p* < 0.05, X * (-) = significant negative effect at *p* < 0.05), X ^0^ = non-significant effect at *p* > 0.05, PA = physical activity, SB = sedentary behavior, BMI = body mass index.

**Table 4:** Intervention effects of community-based interventions (n = 14).

					Outcomes			
Author	Location	N (Age)	Design	Intervention Protocol	PA	SB/Screen Time	BMI	Motor Skills
Barkin et al. (2012)	United States	106 children (M = 4.1 years)	12-week RCT	Parents attended one 90-min group informational session/week Parents were randomly assigned to small social groups			X *	
Bonvin et al. (2013) *	Switzerland	648 children (M = 3.3 years)	9-month RCT	Parents attended informational and discussion sessions led by trained childcare educators	X ^0^		X ^0^	X ^0^
Davison et al. (2011)	United States	422 children (M = 3.4 years)	12-month Pre- post measured (nonpaired quasi experimental)	A community resource guide listed with local outdoor recreation facilities and a community event calendar was sent to parents and updated every 2–3 months	X *	X *		
Fitzgibbon et al. (2013) *	United States	157 children (M = 4.5 years)	14-week RCT	Parents attended a series of 90 min group classes with interactive instruction	X ^0^	X ^0^	X ^0^	
Laukkanen et al. (2017) *	Finland	44 children (M = 6.1 years)	6-month RCT	Parents attended a single 30 min group lecture led by the research team	X *	X *	X ^0^	
Puder et al. (2011) *	Switzerland	652 children (M = 5.2 years)	12-month RCT	Parents attended 3 interactive informational group discussions	X ^0^	X *	X ^0^	X *
Quattrin et al. (2012) *	United States	96 children (M = 4.6 years)	12-week RCT	Parents attended ten 60 min group educational sessions			X *	
Stark et al. (2014) *	United States	18 children (M = 4.6 years)	6-month RCT	Parents attended one 90 min group educational session/week Children would participate in a child group-based session concurrently	X ^0^		X *	
Trost & Brookes (2021) *	Australia	34 children (M = 5.3 years)	8-week RCT	Children and parents were able to share achievements of their app games with family and friends virtually	X ^0^			X *
Tomayko et al. (2016) *	United States	150 children (M = 4.0 years)	24-month RCT	Parents participated in one group meeting/month	X ^0^	X *	X *	
Van de Kolk et al. (2019)	Netherlands	191 children (M = 3.1 years)	3-month Quasi- experimental design	Parents attended three 90 min group seminars A social map indicating PA opportunities was distributed to families	X *	X *	X ^0^	
Walton et al. (2015)	Canada	48 children (M = 3.0 years)	9-week RCT	Parents attended one 2 h group lesson session/week	X ^0^	X ^0^	X ^0^	
Yin et al. (2012)	United States	384 children (M = 4.1 years)	18-week Quasi- experimental pretest/posttest design	Peer educators (parents) delivered 6 educational poster sessions lasting 5–10 min to other parents while they picked up their child from childcare Parents completed a scavenger hunt after the poster session	X *	X ^0^	X *	X *
Zask et al. (2012) *	Australia	560 children (M = 4.5 years)	10-month RCT	Group workshops were held for parents throughout the duration of the intervention			X *	X *

*Notes.* Author citations containing * = those studies are also presented in Table 1. X * = significant positive effect at *p* < 0.05, X ^0^ = non-significant effect at *p* > 0.05, PA = physical activity, SB = sedentary behavior, BMI = body mass index.

**Table 5:** Recommended home- and community-based strategies for early childhood physical activity promotion.

Target Change Outcome: Physical Activity	
Recommended Strategy	Citation
**Distribute Educational Material:** Provide parents with knowledge-based printed (or web-based) materials with information, interactive activities, and suggestions on how to integrate healthy behaviors into home life.	De Craemer et al., 2014; Ray et al., 2020
**Direct Contact with Parents:** Call parents via telephone to discuss barriers, actions, and goals to enhance PA behaviors. Host individual discussions with parents tailored to their family to identify where and how PA may be increased in their daily life.	Laukkanen et al., 2017
**Promote Community Resources:** Provide families with information on physical activity opportunities within their neighborhoods with relevant details (i.e., hours of operation, pictures, parking availability) and a community event calendar through their local neighborhood to encourage physical activity participation with peers.	Davison et al., 2011; Van de Kolk et al., 2019
**Encourage Community Engagement:** Organize group seminars/lectures with parents and families providing education, interactive games, incorporating motivational techniques, and take home activities.	Laukkanen et al., 2017; Van de Kolk et al., 2019
**Peer Education:** Utilize peer educators (other parents willing to be trained) to deliver presentations and group seminars.	Yin et al., 2012

**Table 6:** Recommended home- and community-based strategies for early childhood sedentary behaviors/screen time reduction.

Target Change Outcome: Sedentary Behavior/Screen Time	
Recommended Strategy	Citation
**Home Visits:** Researchers visit the physical homes of families to first observe normal routines/behaviors. After the initial visit, researchers can individually discuss goals with parents and strategize tailored techniques to achieve those goals. Families will also receive culturally sensitive toolkits/materials engrained with knowledge-based content and family activities to do in place of engaging in sedentary behaviors.	Haines et al., 2013; Koulouglioti et al., 2013; Taverno-Ross et al., 2018; Tomayko et al., 2016; Wen et al., 2012
**Distribute Educational Material:** Parents receive either printed, web-based educational materials and activities highlighting sedentary behaviors/screen time and to reinforce already established goals. Parents may also participate in individualized and educational discussions with the research team.	Haines et al., 2013; Keita et al., 2014; Knowlden & Sharma, 2016; Laukkanen et al., 2017; Puder et al., 2011; Tomayko et al., 2016; Wen et al., 2012
**Direct Contact with Parents:** Call and text parents to check-in on progress and to reinforce behaviors and goals previously established. During phone calls, incorporate motivational interviewing to promote behavior change.	Haines et al., 2013; Keita et al., 2014
**Parent Participation:** Parents receive a family exercise video that is conducive for the whole family to participate in. Families also receive a TV monitor that connects to their TVs to restrict screen time.	Keita et al., 2014
**Promote Community Resources:** Provide families with information on physical activity opportunities within their neighborhoods with relevant details (i.e., hours of operation, pictures, parking availability) and a community event calendar through their local neighborhood to encourage physical activity participation with peers.	Davison et al., 2011; Van de Kolk et al., 2019
**Encourage Community Engagement:** Schedule parental group sessions targeting encouragement of PA participation, strategies how parents can role model these behaviors, information on TV use, general parenting tips, and demonstrate co-activities that can be reproduced at home.	Laukkanen et al., 2017; Puder et al., 2011; Van de Kolk et al., 2019

**Table 7:** Recommended home- and community-based strategies for early childhood healthy bmi/weight status.

Target Change Outcome: BMI/Weight Status	
Recommended Strategy	Citation
**Home Visits:** Researchers schedule visits to families’ homes that is incorporates monitoring familial behaviors, setting behavioral goals, monitoring progress, and delivering intervention components (i.e., materials, discussions, activities).	Haines et al., 2013; Haines et al., 2018; Stark et al., 2014; Taverno-Ross et al., 2018; Tomayko et al., 2016; Wen et al., 2012
**Distribute Educational Material:** Provide families with printed, individualized educational materials or tool kits targeting their specific goals previously established with the researchers along with information and strategies targeting PA and other activities they can incorporate in their daily life.	Haines et al., 2013; Latomme et al., 2017; Puder et al., 2011; Wen et al., 2012; Zask et al., 2012
**Direct Contact with Parents:** Parents receive tailored behavior change emails, telephone calls, and text messages specific towards check-ins, reinforcement, and encouragement. One-on-one meetings assisting in behavior goal shaping can be scheduled as needed.	Haines et al., 2013; Haines et al., 2018; Quattrin et al., 2012
**Encourage Community Engagement:** Scheduling group informational sessions focusing on PA promotion, TV use, overall sedentary behavior, FMS promotion, and parenting skills. Parents may also be assigned to social groups throughout the duration of the intervention for enhanced peer support. Children can also participate in group activity sessions with their peers.	Barkin et al., 2012; Puder et al., 2011; Quattrin et al., 2012; Stark et al., 2014; Zask et al., 2012
**Peer Education:** Utilize peer educators (other parents willing to be trained) to deliver presentations and group seminars.	Yin et al., 2012

Notes. BMI = body mass index; FMS = fundamental motor skills.

**Table 8:** Recommended home- and community-based strategies for early childhood motor skill promotion.

Target Change Outcome: Motor Skills	
Recommended Strategy	Citation
**Distributing Educational Material:** Parents receive printed and interactive materials pertaining to overall child health promotion.	Puder et al., 2011; Zask et al., 2012
**Direct Contact with Parents:** Parents download a specific mobile app designed to promote FMS in young children through games and activities, in which parents would receive texts and emails for technological troubleshooting support and to confirm adherence. Parents were also able to share achievements with other family and friends virtually.	Trost & Brookes, 2021
**Community Engagement:** Schedule group discussions and informational activity sessions about PA and sedentary behavior, and how to teach FMS at home.	Puder et al., 2011; Zask et al., 2012
**Peer Education:** Utilize peer educators (other parents willing to be trained) to deliver presentations and group seminars.	Yin et al., 2012

Notes. FMS = fundamental motor skills; PA = physical activity.

## Data Availability

Not applicable.

## Supplementary Materials

The following supporting information can be downloaded at: https://www.mdpi.com/article/10.3390/ijerph191911968/s1, Table S1: PRISMA checklist [75].
